# The diversity and abundance of As(III) oxidizers on root iron plaque is critical for arsenic bioavailability to rice

**DOI:** 10.1038/srep13611

**Published:** 2015-09-01

**Authors:** Min Hu, Fangbai Li, Chuanping Liu, Weijian Wu

**Affiliations:** 1 Guangdong Key Laboratory of Agricultural Environment Pollution Integrated Control, Guangdong Institute of Eco-Environmental and Soil Sciences, Guangzhou 510650, PR China

## Abstract

Iron plaque is a strong adsorbent on rice roots, acting as a barrier to prevent metal uptake by rice. However, the role of root iron plaque microbes in governing metal redox cycling and metal bioavailability is unknown. In this study, the microbial community structure on the iron plaque of rice roots from an arsenic-contaminated paddy soil was explored using high-throughput next-generation sequencing. The microbial composition and diversity of the root iron plaque were significantly different from those of the bulk and rhizosphere soils. Using the *aoxB* gene as an identifying marker, we determined that the arsenite-oxidizing microbiota on the iron plaque was dominated by *Acidovorax* and *Hydrogenophaga*-affiliated bacteria. More importantly, the abundance of arsenite-oxidizing bacteria (AsOB) on the root iron plaque was significantly negatively correlated with the arsenic concentration in the rice root, straw and grain, indicating that the microbes on the iron plaque, particularly the AsOB, were actively catalyzing arsenic transformation and greatly influencing metal uptake by rice. This exploratory research represents a preliminary examination of the microbial community structure of the root iron plaque formed under arsenic pollution and emphasizes the importance of the root iron plaque environment in arsenic biogeochemical cycling compared with the soil-rhizosphere biotope.

Rice is the world’s single most important food crop and a primary food source for more than a third of the world’s population^1^. Among agricultural crops, rice is the major route of exposure for the uptake of inorganic arsenic (As) through food^2^. As toxicity in rice is manifested as reduced growth and sterility and poses potential risks for human exposure through the food chain^3^. Thus, As uptake by rice threatens food security by impacting both the quantity (yield) and quality (toxicity) of rice and represents a serious environmental issue for human health and ecosystem function^4^. The health risk of As in rice is largely based on its inorganic As content because these species are generally considered more toxic than mono- and dimethylarsinic acid^4^. Paddy soil is characterized by a distinct cycle of flooded and non-flooded periods that is accompanied by reduction-oxidation changes. Because the mobility of As is redox-sensitive, this redox change has a significant impact on the behavior of As in the soil particles and pore water of paddy fields, eventually affecting As accumulation in rice plants^5^.

As is readily metabolized by prokaryotes in redox reactions through defensive and respiratory processes^6^. Therefore, As speciation and mobility are affected by microbial metabolism in both aerobic and anaerobic systems. Arsenite-oxidizing bacteria (AsOB) can use As(III) as an electron donor and have evolved multiple pathways for As(III) oxidation to support cell growth^7^. Microorganisms can transform As(III) to less toxic and less mobile As(V) forms; hence, microbial As(III) oxidation has a major impact on the natural attenuation of As pollution by decreasing its bioavailability and removing As from water or soil environments^8^. Bacterial populations are also associated with As(III) oxidation in soil ecosystems^9^,^10^, and many autotrophic or heterotrophic AsOB have been isolated from soil environments^11^,^12^.

Iron plaque, which strongly links soil and plants, is commonly found on the surface of aquatic plant roots due to the release of oxygen and oxidants into the rhizosphere^13^. Due to its highly abundant iron oxyhydroxide deposition (high metal affinity), root iron plaque is a natural adsorbent for toxic elements (As, Cd, Cr, Pb and Ni)^13^,^14^. Oxygen and soluble ferrous iron are key factors in controlling iron plaque generation on the roots of aquatic plants^15^; however, significant numbers of bacteria are associated with iron oxidation within the rhizosphere, which suggests that the microbial community may also play a role in iron plaque formation^16^,^17^. Thus, microbe-generated iron plaque may also impact As speciation at the soil-root interface and reduce As uptake by rice plants.

It has been suggested that As retention is the ultimate mechanism for reducing As mobility on root plaque. However, in anaerobic paddy soil, the dissimilatory reductive dissolution of Fe(III) (hydr)oxides can lead to the release of adsorbed As into the soil aqueous phase^6^. The microbial reduction of As(V) to As(III) also increases the mobility of As due to the lower sorption strength of Fe(III) (hydr)oxides, resulting in the predominance of As(III) among the As species in reducing paddy soil environments. Thus, in As-contaminated flooding soil, anoxic conditions leading to the microbial reduction of As(V) and Fe(III) may enhance the mobility of As, posing a threat to rice plant As transport. Reduced As(III) on soil particles or in solution may be transferred to the root surface and oxidized to As(V) chemically or biologically under anaerobic conditions.

Soil microbes control As transformation reactions that influence As speciation^18^. Rhizosphere soil microbes also play an important role in plant uptake of As from the soil^19^. However, the root iron plaque microbial community is not well characterized, and the role of this community in As uptake by rice should be explored. Furthermore, the population structure and abundance of AsOB on the iron plaque likely plays a significant role in As transformation and decreasing As bioavailability at the soil-plant interface, representing an important influence on As accumulation in rice.

In this exploratory study, next-generation sequencing technologies were applied to resolve the highly complex microbial community structure on the root iron plaque of rice growing in As-affected paddy soil. The taxonomic composition and relative abundance of the AsOB were also investigated using the *aoxB* gene as a functional marker of As(III) oxidizers. We also characterized the differences in microbial composition and diversity among the root iron plaque, rhizosphere soil and bulk soil. Combined with data on As transport in rice roots and the As content in straw and grain, our research provides direct evidence of the effects of the root iron plaque microbiota, particularly the arsenite-oxidizing population, on As uptake by rice.

## Results

### Variations in the soil microbial community structure in the root iron plaque, rhizosphere and bulk soil

The geochemical characteristics of the 15 paddy soils collected from the Lianhuashan mine are presented in Supplementary Table S1. The average pH, organic matter, CEC, and total Si, Ca^2+^, Mg^2+^ and K^+^ values were 6.77, 39.3 g kg^−1^, 18.8 cmol kg^−1^, 27.5 g kg^−1^, 25.6 g kg^−1^, 3.00 g kg^−1^ and 25.3 g kg^−1^, respectively. The total As content of the bulk soils varied from 17.6 to 246.6 mg kg^−1^ with a mean value of 76.0 mg kg^−1^, which is 2.5-fold greater than the maximum allowable concentration (MAC) of total As for agricultural soils in China (30 mg kg^−1^, National Environmental Protection Agency of China GB 15618, 1995). Among these paddy soils, 80% of the samples had As concentrations exceeding the MAC value. The corresponding As content in each part of the rice is given in Supplementary Table S2. The As content in the root ranged from 1.90 to 31.0 mg kg^−1^ with an arithmetic mean of 11.3 mg kg^−1^, whereas the average As content was 0.18 mg kg^−1^ in the grain and 4.45 mg kg^−1^ in the straw.

To explore the diversity and taxonomic composition of the microbial communities in the bulk soil, rhizosphere soil and root iron plaque, molecular biological methods were applied in this study. Genomic DNA was extracted from the above 3 biospheres, and the 16S rRNA genes were sequenced using high-throughput 454 parallel sequencing technology. From the 45 sub-samples (samples of bulk soil, rhizosphere soil and root iron plaque from one site), we obtained a total of 477,897 high-quality sequences with an average read length of 324 bp after quality screening and denoising. The number of sequences per sample ranged from 5,290 to 24,135 with an average of 10,619. When we grouped the sequences into OTUs at the 3% dissimilarity level (roughly corresponding to the species level), the complete dataset included 36,133 OTUs.

**Figure 1:**
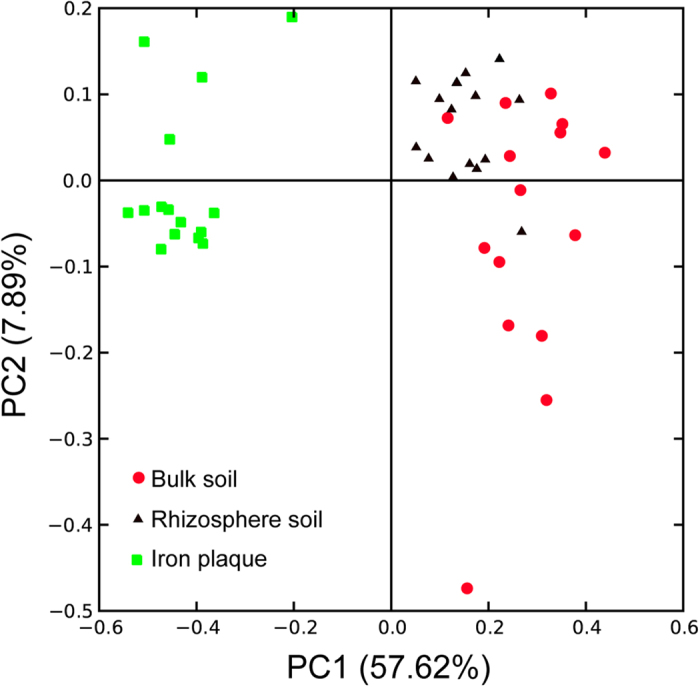
Principal component analysis (PCoA) derived from pairwise unweighted UniFrac distances of 16S rRNA gene between microbial communities of rhizosphere soil (black triangle), bulk soil (red circles) and iron plaque (green squares).

To assess the differences in the structures of the microbial communities between samples, phylogenetic tree-based beta diversity metrics (UniFrac) were calculated. A relatively small UniFrac distance implies that the two communities are similar^20^. Visualization of the unweighted UniFrac distances by PCoA demonstrated that the root iron plaque samples clustered together, whereas the samples from the bulk soil and rhizosphere soil comprised another group (Fig. 1). The unweighted UniFrac distance calculations indicated that the divergence in the microbial community structure of the rhizosphere soil samples was the smallest (with an unweighted UniFrac distance of 0.31 ± 0.06, mean ± SD), whereas these values were 0.45 ± 0.11 for the bulk soil and 0.39 ± 0.11 for the root plaque.

**Figure 2:**
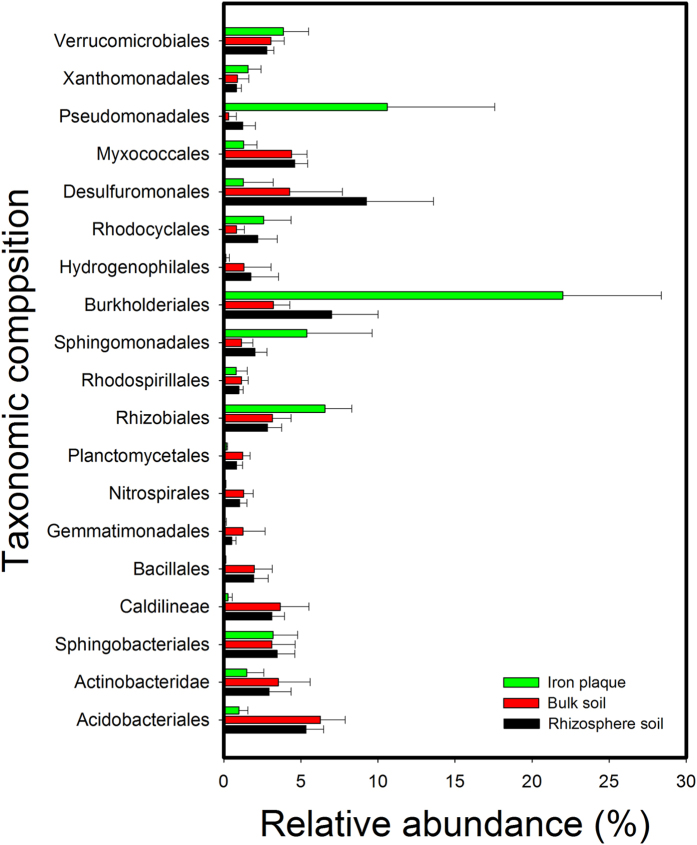
Relative abundance of selected microbial taxa in rhizosphere soil (black columns), bulk soil (red columns) and iron plaque (green columns). Columns and triangles represent average values and error bars give standard errors (n = 15).

To determine which groups of samples or assemblages were similar in species composition, the 16S rRNA gene sequences were assigned to taxonomic groups with 80% confidence or greater (see Supporting Information for details). A comparative analysis of the microbial compositions revealed several large shifts in the relative abundance of many of the dominant phyla in the bulk soil, rhizosphere soil and root iron plaque. The samples from the bulk and rhizosphere soils shared similar order-level profiles, with *Acidobacteriales, Myxococcales* and *Desulfuromonales* most abundant. However, the microbiota of the iron plaque was enriched with *Pseudomonadales, Burkholderiales, Sphingomonadales* and *Rhizobiales* (Fig. 2), in dramatic contrast to the communities of the bulk and rhizosphere soils.

### The correlation between the microbial diversity of root iron plaque and As uptake in rice

To observe the effect of As on microbial community diversity in the bulk soil, rhizosphere soil and iron plaque, we computed Faith’s phylogenetic diversity (PD) and species richness (Chao1) indices by randomly subsampling 5,000 sequences per sample, which avoids the effect of sample size on the microbial community diversity estimation. The PD index considers the degree of relatedness among a set of species in an assemblage, whereas the total species richness (Chao1) includes information on the frequency of rare species in a sample to estimate the number of undetected species in an assemblage^21^. Among the 3 types of sub-samples, the microbial community in the bulk soil exhibited the greatest diversity (average PD and Chao1 were 193 and 5,049, respectively) compared to the rhizosphere soil (average PD and Chao1 were 187 and 4996, respectively) and iron plaque (average PD and Chao1 were 110 and 2615, respectively) (Supplementary Table S3). A one-way analysis of variance (ANOVA) revealed that the microbial diversities (PD and Chao1 indices) of the bulk and rhizosphere soil were significantly higher than that of the iron plaque (all *P* < 0.05) (Supplementary Fig. S1).

As shown in Supplementary Table S4, the total As content of the bulk soil was significantly negatively correlated with the PD and Chao1 metrics of the microbial communities in the bulk soil, rhizosphere soil and iron plaque (all *P* < 0.001), indicating that As decreases both the phylogenetic diversity and species richness of the microbial communities in the soil-root environment. Linear regressions of the Chao1 indices against the As content in the bulk soil were significant and strong (R^2^ = 0.95, 0.88 and 0.71 for bulk soil, rhizosphere soil and iron plaque, respectively. All *P* < 0.001) (Supplementary Fig. S2).

We also evaluated the relationship between the microbial diversity of the root iron plaque and the As content of the rice. The statistical analysis confirmed that the As contents in the rice root, straw and grain were all significantly negatively correlated with the microbial diversity of the iron plaque (Pearson correlation coefficients of −0.934, −0.656 and −0.524 for root, straw and grain, respectively. All *P* < 0.05). Linear regressions between the Chao1 indices of the iron plaque against the rice As concentration were all significant (*P* < 0.001), but the coefficients were as follows: root > straw > grain (R^2^ = 0.70, 0.64, and 0.49, respectively) (Supplementary Fig. S3).

### Abundance and population structure of the arsenite-oxidizing bacteria on the root iron plaque and their relationship with As uptake in rice

**Figure 3:**
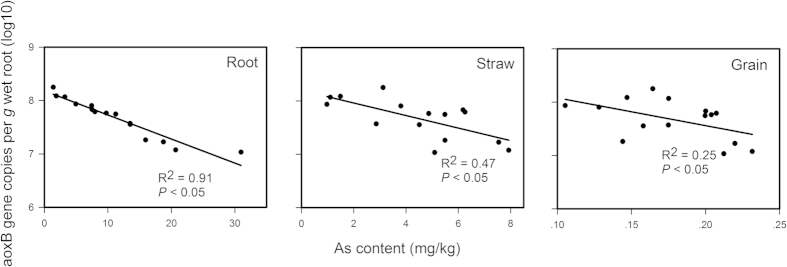
The correlation between abundance of arsenic-oxidizing bacteria (designed as normalized *aox*B gene copy number) on iron plaque and arsenic content in rice root, straw and grain.

**Figure 4:**
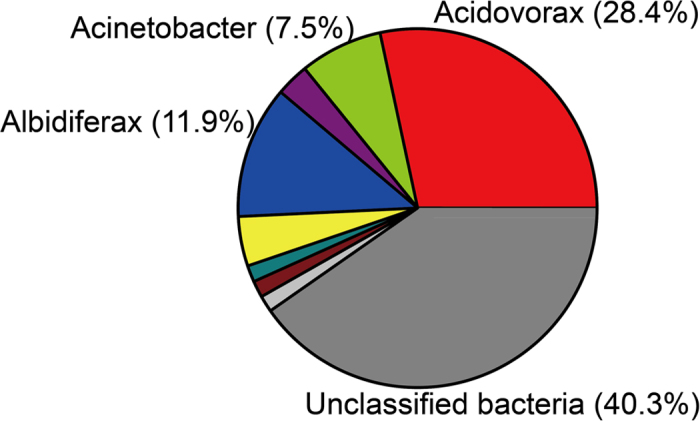
Taxonomic composition of arsenite-oxidizing bacteria of iron plaque at genus level.

Quantification of the number of *aoxB* gene copies using real-time qPCR revealed that the *aoxB* genes of the root iron plaque were present at 1.08 × 10^7^ to 1.78 × 10^8^ copies per g wet roots. The abundance of the *aoxB* gene was significantly negatively correlated with the total As content in the roots and straw (Pearson correlation coefficients of −0.853 and −0.663, respectively; all *P* < 0.001). However, there was no significant correlation between *aoxB* gene abundance and As concentration in the grain (Fig. 3). To obtain insights into the taxonomic composition of the AsOB on the root iron plaque, a clone library was constructed from the DNA extracted from the iron plaque using the *aoxB* gene as a functional marker. A total of 28 different *aoxB* OTUs (97% similarity level) were recovered from 96 sequenced clones. A blastx search of the NCBI-nr database and subsequent phylogenetic inferences from the deduced amino acid sequences revealed that the AsOB on the root iron plaque were dominated by *Acidovorax* (accounting for 28.4% of the total library), unclassified bacteria (22.4%), *Hydrogenophaga* (14.9%) and *Sinorhizobium* (10.4%) (Fig. 4). The most abundant OTU, OTU15, was nearly identical to the *aoxB* amino acid sequences found in two species, *Acidovorax* sp. NO1 (100% amino acid identity) and *Acidovorax* sp. 75 (97% amino acid identity) (Supplementary Fig. S4). The other 5 OTUs that clustered near the *aoxB* gene were from *Albidiferax ferrireducens* T118 (with 91% amino acid identity), which also belongs to the *Comamonadaceae* family. OTU19 was the second most abundant OTU affiliated with the uncultured bacteria. The other *aoxB*-like OTUs were categorized as rare (i.e., OTUs containing less than three representative sequences). Furthermore, many of these rare OTUs formed unique clades and were associated with *Acinetobacter* sp. 33, *Burkholderia vietnamiensis* AU4i, *Ralstonia* sp. 22, *Agrobacterium tumefaciens* and *Aminobacter* sp. 86 (Supplementary Fig. S4).

## Discussion

It is well-accepted that biotic and abiotic As(III) pathways coexist in soil^22^. Biogeochemical processes affecting the behavior of As in the soil environment have been a matter of considerable research interest to determine the chemical or biological factors controlling As(III) oxidation under oxic or anoxic conditions. As(III) is much more slowly oxidized by atmospheric O_2_ than by other components in the soil environment, such as minerals and microorganisms^23^. Owing to their high reactivity at low concentrations and poorly crystalline structures with high surface areas, manganese oxide minerals are thought to be the most important oxidants of abiotic As(III) in nature^24^. In particular, after Fe, Mn is the second most abundant metal element in the root plaques of aquatic plants^25^. Thus, the Mn-induced pathway should be the major abiotic As(III) oxidation route on rice root plaques, although direct experimental evidence for this phenomenon has been lacking. The photocatalytic oxidation of As(III) to As(V) on ferrihydrite is another abiotic pathway for As transformation in nature and has been well researched in high-light water environments^26^. However, photo-induced As(III) oxidation is much less effective in the rhizosphere and deep soil than in the surface layer of soil because of the darkness of such biospheres. Furthermore, a recent report on the redox transformation of arsenic by Fe(II)-activated goethite indicates that As(III) oxidation may occur in the process of Fe(III) oxyhydroxide reduction at the rice root-plaque interface^27^. Kinetic experiments have shown that the rates of the reaction between As(III) and most chemical oxidants are impacted by pH, Eh, adsorbing surfaces, organic matter, and key inorganic substances^28^. During rice growth, the geochemical condition of the paddy soil is influenced by anthropogenic activities, and subsequent changes in the concentrations of O_2_ and Fe species in soil affects abiotic As(III) oxidation on root plaques. Recent microbiological evidence suggests that As(III) is readily oxidized to As(V) by a large diversity of microorganisms under aerobic or anaerobic conditions^6^. Bacterial oxidation of As(III) is typically slower than oxidation via Mn-oxides, but a detailed understanding of the composition and patterns of As(III)-oxidizing microorganisms in rice root plaques is required before we can identify the most important bacterial species and guide the exploration of the potential of these microbes in mitigating health risks associated with arsenic in rice.

Because of its capacity to gradually accumulate metals, root iron plaque has a profound influence on metal uptake and translocation in wetland plants^13^,^14^. Iron plaque is thought to be generated by the excretion of oxygen and oxidants into the rhizosphere by physiologically active rice roots, leading to the oxidization of Fe(II) and the precipitation of Fe(III)^15^. The critical geochemical factors controlling iron plaque formation include radial oxygen loss (ROL), ferrous ion availability, pH and organic carbon^15^,^29^. However, recent studies have revealed that microbial processes are also associated with iron plaque formation on plant roots^16^,^17^. Bacteria such as *Sideroxydans paludicola* and *Sagittaria australis* have been proposed to actively contribute to the formation of iron plaque on plant roots^16^,^17^. The data collected in this study provide the first overview of the iron plaque microbiota under As contamination. We discovered that the root iron plaque of rice was enriched with *Pseudomonadales, Burkholderiales, Sphingomonadales* and *Rhizobiales* (Fig. 2). This detailed information about the community structure of microbes on iron plaque will aid in understanding and predicting Fe-As element biogeochemical cycling in the micro-biosphere. The UniFrac PCoA clustering results suggested that the microbial community structure of the iron plaque was significantly distinct from that of the bulk and rhizosphere soils (Fig. 1). Accumulating evidence suggests that long-term As exposure permanently alters the microbial community structures of the bulk and rhizosphere soils^16^,^17^,^30^,^31^ by decreasing the alkaline phosphatase, arylsulfatase, protease and urease activities of soil microorganisms^32^. Contamination was observed to impact microbial diversity and species richness in As-contaminated soils^33^. In our study, the microbial community diversity was significantly negatively correlated with the As contents of the bulk soil, rhizosphere soil and iron plaque (Supplementary Fig. S2), indicating that As reduces microbial diversity not only in the soil-rhizosphere ecosystem but also at the soil-root interface. In addition, microbial diversity was significantly lower in the iron plaque than in the bulk and rhizosphere soils (Supplementary Fig. S1). First, this observation may be a result of the high iron content and micro-aerobic conditions of the root iron plaque. Second, during Fe(II) oxidation on the iron plaque, the protons that are generated and released reduce the pH at the root surface^34^, thus likely decreasing the phylogenetic diversity and structure of the microbial communities. A comparative analysis of the heavily metal-contaminated bulk and rhizosphere soils of the metal-hyperaccumulating plant *Thlaspi caerulescens* revealed similar microbial community structures and diversities^35^. This result is consistent with our study of the microbial communities of As-contaminated bulk and rhizosphere soils. In this study, the distances within the microbial community of the rhizosphere soil (with an average unweighted UniFrac distance of 0.31) were shorter than those of the bulk soil and iron plaque communities, suggesting that under As pollution, the microbial community in the rhizosphere was less diverse than those of the bulk soil and iron plaque.

Much attention has been paid to microbial As oxidation in aquatic or soil environments. However, the role of AsOB at the soil-plant interface (root iron plaque) has not been explored. As a phylogenetic maker, the *aoxB* gene is widely used for studying the abundance and taxonomy of AsOB in nature. It has been estimated that there are as many as 1.7 × 10^7^ copies of *aoxB*-related genes per g of As-amended soil^33^; because AsOB typically contain only one or two copies of the *aoxB* gene in their genomes^36^, this finding represents approximately 10^7^ AsOB per g of As-contaminated soil. In our study, the number of *aoxB* gene copies retrieved from 1 g of wet roots ranged from 1.08 × 10^7^ to 1.78 × 10^8^. The high number of *aoxB* gene copies indicates the As(III)-oxidizing potential of the microorganisms on the rice root plaque. Because of the difficulty in identifying As species on the root plaque, further studies should be performed to verify the microbes that mediate As(III)-oxidizing processes on the root plaque using X-ray absorption near edge structure (XANES) technology combined with molecular biology. The rice rhizosphere is highly oxygenated, and most AsOB isolated from soil are aerobes that utilize O_2_ as an electron acceptor^6^. Thus, aerobic microbial oxidation of As(III) may occur readily and rapidly on the root plaque. Under anaerobic conditions, the As(V) attached to soil minerals is readily reduced to As(III)^37^, increasing As mobility in the soil biotope. Then, the reduced As(III) may move to the rhizosphere and become oxidized on the root plaque, resulting in decreased As bioavailability for the rice root. Moreover, arsenite and arsenate are taken up into plant roots by different mechanisms. Arsenate is taken up by phosphate transporters, whereas arsenite is taken up by rice roots mainly through the Si uptake pathway^38^. Thus, the As(III)-oxidizing bacteria on the root plaque may impact the pathway of As uptake by the rice root. Therefore, aerobic As(III) oxidation on the root plaque is more effective than that in soil under anaerobic flooding conditions. Thus, it is evident that biological As(III) oxidation occurring at the root plaque interface is closely intertwined with As uptake by rice.

Compared with aerobic As(III)-oxidizing microorganisms, little is known about anoxic As(III)-oxidizers. Although oxidation with O_2_ is more favorable based on biochemical energetic considerations, alternative oxidants with lower reduction potentials are feasible for the oxidation of As(III). 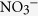, which has a higher electrochemical potential than As(III) under standard conditions, can be an alternate electron acceptor to support the oxidation of As(III) to As(V) by denitrifying bacteria under anoxic conditions. Evidence is growing that the anoxic oxidation of arsenite linked to nitrate reduction is feasible in continuous bioreactors^39^, sludges and sediments^40^. However, As(III) oxidation coupled to nitrate reduction has not been reported in paddy soil. In the denitrification zone (always below 3 mm in soil depth), nitrate is produced by ammonium transformation during fertilization with chemical fertilizers such as ammonium sulfate or organic fertilizers such as urea and straw^41^. In such an oxygen-depleted zone, nitrate replaces oxygen as the major electron acceptor for As(III) oxidation, and this zone also provides space for microorganisms capable of arsenite oxidation coupled to nitrate reduction to survive in paddy soil. It has been shown that the addition of nitrate results in decreased As accumulation by rice, suggesting a possible link between nitrate reduction and As(III) oxidation in paddy soil^42^. From an arsenic-contaminated paddy soil, a newly anaerobic, autotrophic As(III)-oxidizing bacterium was isolated that also exhibited the ability to reduce nitrate^12^. Furthermore, our recent data shows that As(III) oxidation is promoted by nitrate addition (data not published). Heterotrophic As(III) oxidation is generally assumed to be a detoxification process in which the microorganisms do not obtain energy from the oxidation of As(III)^8^. Thus, the anoxic oxidation of As(III) linked to nitrate reduction should be a mechanism for energy generation coupled with metal detoxification. In future, the microbial composition of nitrate reducers and As(III) oxidizers should be explored using the *narG* and *aoxB* genes as phylogenetic makers, respectively, to identify the underlying microbial mechanisms. Additionally, the relevance of As(III) oxidation and nitrate reduction in the root plaque system remains unknown and must be determined through future studies.

Recently, it has been shown that the Fe(III) oxides generated by nitrate-dependent, Fe(II)-oxidizing bacteria are strongly adsorbing for As(V)^43^. Thus, the simultaneous microbial oxidation of Fe(II) and As(III) facilitated by nitrate may be a significant process leading to the formation of particulate ferric-oxide and As(V), resulting in immobilized As in the form of As(V) adsorbed onto biogenic Fe(III) (hydr)oxides with reduced mobility and toxicity in the rice root plaque. We identified *Acidovorax, Hydrogenophaga* and *Sinorhizobium* as the major genera of the AsOB on the iron plaque of paddy rice (Fig. 4). The most abundant *aoxB* sequences were affiliated with *Acidovorax* sp. strain NO1 and *Albidiferax ferrireducens* T118, which belong to the Comamonadaceae family. *Acidovorax* sp. strain NO1 is a facultative, anaerobic, arsenite-oxidizing and nitrate-reducing bacterium that was isolated from gold mine soil^44^, whereas *Albidiferax ferrireducens* (formerly known as *Rhodoferax ferrireducens*) strain T118 is a dissimilatory iron-reducing bacterium used as an acetate electron acceptor^45^. The *Acidovorax* genus harbors many typical strains of neutrophilic FeOB, such as *Acidovorax* sp. strains BoFeN1 and 2AN and *Acidovorax ebreus* strain TPSY^46^,^47^,^48^. These results indicate that the biological process of As oxidation is tightly coupled with iron cycling; in fact, Fe-As coupling oxidation may occur at the same genus level or even on the strain level, although experimental evidence is lacking to support the hypothesis that neutrophilic FeOB are capable of As(III) oxidation. This lack of evidence is largely because research on microbial As(III) oxidation in neutral pH environments has focused on sediment or surface water ecosystems rather than anaerobic or micro-aerobic soil or even As-contaminated soil ecosystems. Prior studies have only revealed that the redox cycling of iron affects the release, transport, immobilization and bioavailability of As in paddy soils^5^. The relationship between microbial dissimilatory Fe(III) reduction and As(V) reduction has been well studied in model organisms such as *Shewanella putrefaciens* strain CN-32^49^. However, the coupling of Fe(II) oxidation with As(III) oxidation in a soil environment is not as well understood. Our research on iron plaque suggests that *Acidovorax*-affiliated strains may be the ideal microorganism for investigating the role of bacterially induced, As(III)- and Fe(II)-coupled oxidation in controlling As mobility in soil ecosystems. Due to the higher As and Fe concentrations of rice iron plaque in As-polluted paddy soil, more effort is needed to isolate strains capable of As(III) and Fe(II) oxidation from such biospheres.

A synthesis of the available data suggests that rhizo-bacteria reduce metal uptake in metal-tolerant plants by accumulating heavy metals in the soil in a plant-unavailable form. However, many metal-resistant bacteria can promote the uptake of heavy metals in plants by increasing water-soluble metals in the soil solution^50^. The above evidence suggests that the type of microbes and the metal uptake capacity of plants are two factors in plant-microbe interactions that are affected by metal contamination. There is no doubt that the biogeochemical cycling of As in bulk soil or the rhizosphere interface affects As mobility in soil and its bioavailability to the plant^19^. However, element accumulation and speciation in the iron plaque at the root surface is pivotal in understanding the transfer of nutrients or contaminants into rice roots. In addition, As distribution and speciation near rice roots is influenced by iron plaque and the redox conditions of the soil matrix^51^. To address the effects of AsOB on As uptake in rice, we should consider the iron plaque because of the strong association of Fe-As element cycling with root plaque and because iron plaque is the last barrier to toxic elements in rice roots. The higher dependence of the As content in rice roots on the abundance of AsOB on iron plaque may be a result of the co-evolution of the plant and microbes under metal stresses. The oxygen released from the rice roots affects the As-oxidizing population density and activity and also promotes the formation of iron plaque, which adversely leads to As immobilization on the root surface and reduces As bioavailability to the rice plant.

The oxidized species, As(V), is much more strongly adsorbed by the iron plaque than As(III), which eventually reduces As bioavailability^52^. This process is in agreement with the finding that much of the adsorbed As on the rice iron plaque appears to be arsenate^13^, although both arsenate and arsenite were found to be present in association with the iron plaque of other wetland plants^53^. The formation of root iron plaque is promoted by the release of oxygen and oxidants into the rhizosphere^13^. However, recent studies have indicated that the microbial community associated with the root iron plaques of wetland plants are enriched in iron-oxidizing bacteria^16^,^54^, suggesting the key role of this activity in the root plaque microbial community. Therefore, As(III) may be transferred from soil particles or solution to the rice root plaque and oxidized to As(V) by microorganisms, which thus contributes an additional strategy for improving As immobilization and mitigating As contamination in rice. Our study demonstrates a significant correlation between the abundance of As(III)-oxidizing bacteria and As content in rice root, suggesting another pathway for decreasing As bioavailability through the rice root plaque microbial community.

## Methods

### Root iron plaque extraction and microbial community structure analysis

The rice roots were washed with distilled water more than three times to remove the soil particles adhering to the root surface and were then rinsed with sterile water. The iron plaque was then extracted from the root materials using a dithionite-citrate-bicarbonate (DCB) solution as described previously^13^, and the DCB-extract solution was centrifuged at 16,000 × g for 10 min to pellet any microorganisms present on the plaque. The genomic DNA was extracted from the precipitated products of the DCB extract (iron plaque), bulk soil and rhizosphere soil using the PowerSoil^TM^ DNA Isolation Kit (MO BIO Laboratories, Inc., Carlsbad, CA) according to the manufacturer’s instructions. The details of the 16S rRNA gene PCR, 454 pyrotag sequencing and bioinformatic analysis are presented in the Supporting Information.

### Clone library and qPCR of the *aoxB* gene from the root iron plaque

The *aoxB* genes (encoding the As(III) oxidase catalytic subunit) were recovered using universal *aoxB* gene primers as described previously^55^. Quantitative real-time polymerase chain reaction (qPCR) assays of the *aoxB* genes used the primers M1-2F (5′-CCA CTT CTG CAT CGT GGG NTG YGG NTA-3′) and M2-1R (5′-GGA GTT GTA GGC GGG CCK RTT RTG DAT-3′)^56^. Details of the amplification conditions and the *aoxB* sequence analysis are in the Supporting Information.

### Statistical analyses

Correlations between microbial community diversity and environmental variables were estimated using Pearson correlation coefficients using the statistical software package SYSTAT 18 (SPSS, Inc., Chicago, IL, USA). The curve fitting feature of SigmaPlot (Jandel Scientific, San Jose, CA, USA) was used to fit the data points for microbial diversity (Chao1) or normalized *aoxB* gene copy numbers of the iron plaque against the As content of the different components of mature rice plants (root, straw, and grain).

### Sequence accession

The 16S rRNA gene sequences reported in this paper have been deposited in the National Center for Biotechnology Information Sequence Read Archive with accession No. SRP045799, and the nucleotide sequences of the *aoxB* genes were also deposited in GenBank under the accession numbers KM355781-KM355834.

Further details on the methods used in this study are included in the Supporting Information online.

## Additional Information

**How to cite this article**: Hu, M. *et al.* The diversity and abundance of As(III) oxidizers on root iron plaque is critical for arsenic bioavailability to rice. *Sci. Rep.* **5**, 13611; doi: 10.1038/srep13611 (2015).

## Supplementary Material

Supplementary Information
