# Supplementary material for: The diversity and abundance of As(III) oxidizers on root iron plaque is critical for arsenic bioavailability to rice
Source: Sci Rep. 2015 Sep 1;5:13611. doi: 10.1038/srep13611 (PMC4555042; doi:10.1038/srep13611)
Supplement: Supplementary Information [file srep13611-s1.doc]

**SUPPORTING INFORMATION**

**For**

The diversity and abundance of As(III) oxidizers on root iron plaque is critical for arsenic bioavailability to rice

Min Hu^1^, Fangbai Li1[[1]](#footnote-2)*, Chuanping Liu^1^ and Weijian Wu^1^

^1^ *Guangdong Key Laboratory of Agricultural Environment Pollution Integrated Control,* *Guangdong Institute of Eco-Environmental and Soil Sciences, Guangzhou 510650, PR China*

**SUPPLEMENTARY METHODS**

**Sampling and Geochemical Analysis**

The Lianhuashan tungsten mine, located in Shantou City, Guangdong Province (23°38′30.4″N, 116°50′4.7″E), was once one of the largest tungsten mines in southern China.^1^ The mining operation was closed in 1991, and huge amounts of abandoned tailings were left behind. Acid mine drainages (AMDs) from the mine tailing often flood nearby surrounding areas in the rainy season, leading to the deposition of huge amounts of heavy metals in the farmlands. We previously demonstrated that As was the most important metal contaminant in environmental samples from these areas.^1, 2^

In October 2012, paddy soil and rice (*Oryza sativa* L.) samples were collected from farmland surrounding the Lianhuashan tungsten mining area. The bulk soil samples were collected from the top 0-20 cm of soil. Furthermore, the roots, straw and grains of rice were collected from the field before harvest at maturity. The soil samples for the biological analyses were transferred to sterile 50-mL polypropylene tubes and shipped on ice to the laboratory, where they were stored at -40 °C. The soil properties, including pH, OM (organic matter), CEC (cation exchange capacity), N (total nitrogen), and total Si, Ca^2+^, Mg^2+^, and K^+^, were determined for each sample as described previously[^1^](#_ENREF_1). The total As content of the bulk soil, roots, straw, and grains of rice was analyzed as described previously.

**DNA Extraction, 16S *rRNA* PCR and 454 Pyrotag Sequencing**

Genomic DNA was extracted from the precipitation products of the DCB extract (iron plaque), bulk soil and rhizosphere soil using a PowerSoil^TM^ DNA Isolation Kit (MO BIO Laboratories, Inc., Carlsbad, CA) according to the manufacturer’s instructions. The genomic DNA extraction was performed in triplicate for each sample. The DNA was eluted using 100 μL of TE buffer (10 mmol L^-1^ Tris-HCl (8.0), 1 mmol L^-1^ EDTA) and run on a 1% agarose gel. The quantity of the eluted DNA was estimated using a Qubit® dsDNA BR (Broad Range) Assay Kit (Invitrogen, Merelbeke, Belgium) in combination with a Qubit® 2.0 Fluorometer (Invitrogen, Merelbeke, Belgium). The quantified DNA was stored at -80 °C until downstream molecular analysis.

The analysis of the 16S *rRNA* genes was performed as previously described[^4^](#_ENREF_4). Briefly, a set of two eubacterial primers, 515F (5’- GTG CCA GCM GCC GCG GTA A-3’) and 806R (5’-GGA CTA CVS GGG TAT CTA AT-3’), was designed to amplify the hypervariable V3-V4 region of the 16S *rRNA* gene from nearly all eubacteria. The forward primer contained the Titanium A adaptor, and the reverse primer contained the Titanium B adaptor. To allow sample multiplexing during pyrosequencing 8-bp Hamming barcodes were added to the 5’ end of primer 515F. Three replicates of the DNA extract from each sample were amplified by PCR. PCR was conducted using a PCR thermal cycler Model C1000 (Bio-Rad, Richmond, CA). The total volume of the reaction mixture was 50 μL, which consisted of 0.5 μL of each primer (50 pmol each), 5 μL of 2.5 mmol L^-1^ dNTP mixture, 5 μL of 10× *Ex* Taq buffer (20 mmol L^-1^ Mg^2+^; TaKaRa Inc., Dalian, China), 0.25 μL of *Ex* Taq DNA polymerase (TaKaRa), 1 μL of the environmental DNA template and 37.75 μL of Milli-Q water. The cycle conditions for the PCR amplification were as follows: initial denaturation at 94 °C for 3 min, followed by 30 cycles of denaturation at 94 °C for 30 s, annealing at 50 °C for 30 s and extension at 72°C for 30 s, and an extension step at 72 °C for 5 min after cycling was complete. All samples were amplified in triplicate, and no-template controls were included in all steps of the process. Next, 5 μL of each reaction mixture was analyzed on a 2% agarose gel, and the PCR products were gel-purified using a QIAquick Gel Extraction Kit (Qiagen). The purified PCR amplicons were combined in equimolar ratios into a single tube after the concentration of each amplicon was determined using the Quant-iT PicoGreen dsDNA reagent kit (Life Technologies, Merelbeke, Belgium); they were then pooled in equimolar amounts, vacuum dried, and sequenced using a 454 Life Sciences Genome Sequencer FLX (Roche Diagnostics, Indianapolis, IN, USA) machine with Titanium Chemistry at Macrogen Inc. (http://www.macrogen.com, Seoul, South Korea).

**Bioinformatics Analyses of the 16S *rRNA* Amplicons**

All 454 community 16S *rRNA* sequences were denoised (homopolymer error-correction) using Denoiser V0.91 software[^5^](#_ENREF_5) according to the manual. Using QIIME-1.5.0,^5^ short reads were removed, and only sequences longer than 200 bp were included in the downstream analysis; low-quality reads were also removed from the analysis using the default settings (average quality value of > 20). In addition, sequences containing > 6 nt ambiguous nucleotides (‘‘N’’) in the homopolymeric regions were removed. The sequences were then assigned to each sample with an 8-bp barcode using a script derived from the QIIME pipeline. Second, the remaining sequences from all samples were clustered into Operational Taxonomic Units (OTUs) at 97% sequence similarity with an “uclust” model (search and clustering orders of magnitude faster than BLAST). The representative sequences in each OTU were assigned to taxonomic groups using the RDP classifier[^6^](#_ENREF_6) within an 80% confidence threshold. Finally, to estimate alpha diversity, a random sub-sampling method for each sequence library was used for microbial community diversity index calculations to control for the effects of library size. Alpha diversity index (Chao1 and PD) analyses were performed for all samples with 100 repetitions using a step size of 5,000 sequences per sample. For beta diversity analysis, all samples were also subsampled to 5,000 sequences per sample to remove all possible side effects of sample size. Principal coordinate analysis (PCoA) was performed on pairwise unweighted UniFrac distances[^7^](#_ENREF_7) using the QIIME software package.

**Clone Library and *q*PCR of the *aox*B Gene of Root Iron Plaque**

The *aox*B genes (encoding the As(III) oxidase catalytic subunit) were recovered using universal *aox*B gene primers as described previously[^8^](#_ENREF_8). Briefly, the primers M1-2F (5’-CCA CTT CTG CAT CGT GGG NTG YGG NTA-3’) and M3-2R (5’-TGT CGT TGC CCC AGA TGA DNC CYT TYT C-3’) were used to amplify the partial sequence of the *aox*B gene. The amplification conditions were as follows: an initial denaturation at 94 °C for 2 min, followed by 28 cycles of denaturation at 94 °C for 30 s, annealing at 63 °C for 30 s and extension at 72 °C for 30 s, and an extension step at 72°C for 5 min after cycling was complete. All samples were amplified in triplicate, and no-template controls were included in all steps of the process. The resulting PCR products were separated by electrophoresis (1.5% agarose), and the products of the expected size (~1100 bp) were excised from the gel and eluted with a Gel Extraction Kit (Omega Bio-Tek, Doraville, CA). The purified fragments were cloned with a pGEM-T cloning kit for sequencing (Promega, Madison, WI) according to the manufacturer’s instructions. The colonies were screened via PCR using SP6/T7 primers to determine the insert size, and 96 clones of the expected size were sequenced on ABI 3730XL capillary sequencing machines. Sequences containing > 10 nt ambiguous nucleotides (‘‘N’’) or Q20 bases (99% accuracy) < 90% sequence length were removed from the downstream analysis. The high-quality sequences were then assigned to taxonomic groups by blast analysis against the NCBI-nr database and comparison with the Functional Gene Pipeline/Repository[^9^](#_ENREF_9) within a 90% confidence threshold. Sequences with a poor sequence alignment, no BLAST hits, or BLAST hits to genes that did not encode *aox*B were removed from the downstream analyses. The *aox*B gene sequences containing the reference sequences with high identity (downloaded from NCBI or the Functional Gene Pipeline/Repository) were translated into amino acid sequences. Phylogenetic trees of the translated amino acid sequences were then constructed in MEGA6[^10^](#_ENREF_10) using default parameters based on the neighbor-joining method with 1,000 bootstrap replicates.

Quantitative real-time polymerase chain reaction (qPCR) assays of the *aox*B genes were prepared in quadruplicate with 1 μL of diluted DNA (0.1 ng mL^-1^) in a final volume of 25 μL using iQ SYBR Green Supermix (Bio-Rad) with the primers M1-2F (5’-CCA CTT CTG CAT CGT GGG NTG YGG NTA-3’) and M2-1R (5’-GGA GTT GTA GGC GGG CCK RTT RTG DAT-3’)[^11^](#_ENREF_11). One *aox*B-containing plasmid from the clone library was used as a positive control and to create standard curves for the qPCR assays. All qPCR reactions were prepared in quadruplicate using the Bio-Rad iQ5^TM^ system. The qPCR conditions consisted of an initial denaturation step at 95 °C for 5 min, followed by 40 cycles of denaturation at 95 °C for 15 s, annealing at 60 °C for 45 s and extension at 72 °C for 30 s, and an extension step at 72°C for 5 min after cycling was complete. A final dissociation step was performed at 65-95°C. Previously, the gene copy number was normalized to micrograms of DNA or grams of soil to represent the relative abundance of the genes present in the soil environment[^12^](#_ENREF_12). However, in the present study, the AsOB abundance on root iron plaques was more related to the surface area or the biomass of the rice root. Therefore, we normalized the copy number of the *aox*B gene molecules to the wet weight of the rice roots to indicate the density of AsOB on root iron plaques.

1. Liu, C.P. *et al.* Arsenic contamination and potential health risk implications at an abandoned tungsten mine, southern China. *Environ Pollut* **158**, 820-826 (2010).

2. Liu, C.P. *et al.* Effects of calcium peroxide on arsenic uptake by celery (*Apium graveolens* L.) grown in arsenic contaminated soil. *Chemosphere* **86**, 1106-1111 (2012).

3. Liu, W.J. *et al.* Arsenic sequestration in iron plaque, its accumulation and speciation in mature rice plants (*Oryza Sativa* L.). *Environ Sci Technol* **40**, 5730-5736 (2006).

4. Peiffer, J.A. *et al.* Diversity and heritability of the maize rhizosphere microbiome under field conditions. *Proc Natl Acad Sci U S A* **110**, 6548-6553 (2013).

5. Reeder, J. & Knight, R. Rapidly denoising pyrosequencing amplicon reads by exploiting rank-abundance distributions. *Nat. Methods* **7**, 668-669 (2010).

6. Cole, J.R. *et al.* The Ribosomal Database Project: Improved alignments and new tools for rRNA analysis. *Nucleic Acids Res.* **37**, D141-D145 (2009).

7. Lozupone, C., Lladser, M.E., Knights, D., Stombaugh, J. & Knight, R. UniFrac: an effective distance metric for microbial community comparison. *ISME J.* **5**, 169-172 (2011).

8. Quéméneur, M. *et al.* Diversity surveys and evolutionary relationships of *aox*B genes in aerobic arsenite-oxidizing bacteria. *Appl Environ Microbiol* **74**, 4567-4573 (2008).

9. Fish, J.A. *et al.* FunGene: the functional gene pipeline and repository. *Front Microbiol* **4**, 291 (2013).

10. Tamura, K., Dudley, J., Nei, M. & Kumar, S. MEGA4: molecular evolutionary genetics analysis (MEGA) software version 4.0. *Mol Biol Evol* **24**, 1596-1599 (2007).

11. Quéméneur, M. *et al.* Population structure and abundance of arsenite-oxidizing bacteria along an arsenic pollution gradient in waters of the Upper Isle river basin, France. *Appl Environ Microbiol* **76**, 4566-4570 (2010).

12. Saleh-Lakha, S. *et al.* Microbial gene expression in soil: methods, applications and challenges. *J Microbiol Methods* **63**, 1-19 (2005).

**Table S1.** Physical–chemical characteristics of soils of LHM paddy soil (Guangdong, southern China) ^*^.

| Sampling site | pH | OM (g kg^-1^) | CEC | N (g kg^-1^) | Si | Ca^2+^ | Mg^2+^ | K^+^ |
| --- | --- | --- | --- | --- | --- | --- | --- | --- |
| SY01 | 6.62 | 25.7 | 15.7 | 1.76 | 72.3 | 21.3 | 1.77 | 16.0 |
| SY02 | 6.44 | 31.6 | 15.9 | 1.57 | 34.8 | 16.7 | 1.11 | 15.7 |
| SY03 | 6.55 | 25.5 | 13.1 | 1.29 | 28.9 | 14.6 | 1.34 | 17.0 |
| SY04 | 6.63 | 28.8 | 15.2 | 1.61 | 36.2 | 15.7 | 1.92 | 17.2 |
| SY05 | 6.75 | 27.8 | 12.0 | 1.51 | 70.9 | 18.5 | 1.64 | 16.1 |
| SY06 | 6.73 | 23.9 | 11.9 | 1.43 | 24.7 | 14.0 | 0.95 | 19.6 |
| SY07 | 6.65 | 29.6 | 16.2 | 1.70 | 47.7 | 17.3 | 1.60 | 19.8 |
| SY08 | 6.69 | 39.3 | 17.7 | 2.04 | 52.5 | 18.4 | 2.31 | 20.3 |
| SY09 | 6.80 | 35.3 | 18.8 | 2.27 | 49.2 | 17.2 | 3.00 | 20.6 |
| SY10 | 7.00 | 22.1 | 14.1 | 1.62 | 55.6 | 19.5 | 1.55 | 20.3 |
| SY11 | 6.95 | 36.7 | 17.6 | 2.21 | 51.4 | 18.4 | 1.68 | 18.3 |
| SY12 | 7.02 | 24.2 | 16.2 | 1.33 | 47.7 | 15.0 | 2.43 | 19.3 |
| SY13 | 6.56 | 17.2 | 13.0 | 1.08 | 57.3 | 14.2 | 1.81 | 21.4 |
| SY14 | 6.95 | 23.3 | 14.7 | 1.32 | 37.8 | 25.6 | 1.20 | 20.1 |
| SY15 | 7.15 | 21.7 | 11.1 | 1.21 | 61.3 | 14.2 | 1.57 | 25.3 |
| mean | 6.77 | 27.5 | 14.9 | 1.60 | 48.6 | 17.4 | 1.73 | 19.1 |

*OM: Organic matter content, OC: Organic, carbon content; CEC: Cation exchange capacity (cmol+kg-1)

**Table S2.** As concentration in paddy soil or rice form Lianhuashan Mountain area (Guangdong southern China, mg kg^-1^ dry matter, n=15)^a^

| Site | As contention (mg/kg) | | | | |
| --- | --- | --- | --- | --- | --- |
|  | Paddy soil ^b^ |  | Rice ^c^ | | |
|  |  |  | Root | Straw | Grain |
| SY01 | 246.6 |  | 31.0 | 5.09 | 0.21 |
| SY02 | 101.9 |  | 16.0 | 5.49 | 0.14 |
| SY03 | 149.8 |  | 20.7 | 7.93 | 0.23 |
| SY04 | 115.8 |  | 18.8 | 7.54 | 0.22 |
| SY05 | 73.5 |  | 13.5 | 2.86 | 0.17 |
| SY06 | 51.7 |  | 7.97 | 6.26 | 0.21 |
| SY07 | 94.4 |  | 13.5 | 4.51 | 0.16 |
| SY08 | 54.2 |  | 9.77 | 4.87 | 0.20 |
| SY09 | 37.2 |  | 7.45 | 3.80 | 0.13 |
| SY10 | 17.6 |  | 1.41 | 3.12 | 0.16 |
| SY11 | 40.4 |  | 7.52 | 6.18 | 0.20 |
| SY12 | 29.4 |  | 3.24 | 1.11 | 0.18 |
| SY13 | 26.6 |  | 1.90 | 1.49 | 0.15 |
| SY14 | 36.2 |  | 4.97 | 0.98 | 0.11 |
| SY15 | 64.2 |  | 11.3 | 5.49 | 0.20 |

^a^ The soils were air-dried and sieved to < 2 mm before metals determination;

^b^ Maximum allowable concentration of As in agricultural soil is 30 mg/kg according to National Environmental Protection Agency of China, GB15618, 1995;

^c^ Maximum permissible limits of As in rice is 0.5 mg/kg according to SEPA, 2005.

**Table S3.** Alpha diversity indices of microbial community ^a^

| Sites | Bulk soil | |  | Rhizosphere soil | |  | Iron plaque | |
| --- | --- | --- | --- | --- | --- | --- | --- | --- |
|  | PD^b^ | chao1 |  | PD | chao1 |  | PD | chao1 |
| SY01 | 166 | 3643 |  | 158 | 4326 |  | 80 | 1839 |
| SY02 | 170 | 3872 |  | 174 | 3794 |  | 99 | 2330 |
| SY03 | 166 | 4045 |  | 164 | 4127 |  | 89 | 1725 |
| SY04 | 168 | 4238 |  | 171 | 4200 |  | 99 | 2261 |
| SY05 | 188 | 4321 |  | 188 | 4609 |  | 110 | 2804 |
| SY06 | 194 | 5231 |  | 191 | 5385 |  | 112 | 2217 |
| SY07 | 186 | 4364 |  | 183 | 4500 |  | 108 | 2651 |
| SY08 | 178 | 4875 |  | 183 | 4893 |  | 103 | 2315 |
| SY09 | 206 | 5831 |  | 195 | 5447 |  | 117 | 2884 |
| SY10 | 238 | 7036 |  | 208 | 6125 |  | 140 | 3098 |
| SY11 | 202 | 5864 |  | 193 | 5258 |  | 112 | 2736 |
| SY12 | 218 | 5657 |  | 205 | 5536 |  | 124 | 3006 |
| SY13 | 219 | 6320 |  | 207 | 6403 |  | 126 | 3494 |
| SY14 | 213 | 5642 |  | 201 | 5828 |  | 117 | 3089 |
| SY15 | 187 | 4799 |  | 185 | 4512 |  | 110 | 2782 |

^a^ All samples were also subsampled to 5,000 sequences and OTUs were clustered at 97% similarity before indices calculation;

^b^ PD: phylogenetic distance.

**Table S4.** The correlations between microbial community diversity and As concentration in paddy soil or rice.

| Microbial community diversity | | As content | | | |
| --- | --- | --- | --- | --- | --- |
|  |  | Soil | Rice Root | Rice Straw | Rice Seed |
| Bulk soil | PD | **-.783**** | **-.879**** | **-.722**** | **-.534*** |
|  | chao1 | **-.809**** | **-.888**** | **-.546*** | -0.408 |
|  |  |  |  |  |  |
| Rhizosphere soil | PD | **-.911**** | **-.966**** | **-.742**** | **-.565*** |
|  | chao1 | **-.714**** | **-.829**** | **-.691**** | -0.444 |
|  |  |  |  |  |  |
| Root plaque | PD | **-.885**** | **-.934**** | **-.656**** | **-.524*** |
|  | chao1 | **-.793**** | **-.835**** | **-.802**** | **-.684**** |

**SUPPLEMENTARY FIGURES CAPTIONS**

**Figure S1.** The microbial community diversity of bulk soil, rhizosphere soil and iron plaque.

**Figure S2.** The correlation between total As content in bulk and microbial community diversity of bulk soil, rhizosphere soil and iron plaque.

**Figure S3.** The correlation between microbial community diversity of iron plaque and arsenic content in rice root, straw and grain.

**Figure S4.** Neighbor-joining tree showing the phylogenetic relationship of arsenite-oxidizing bacteria on the root iron plaque.


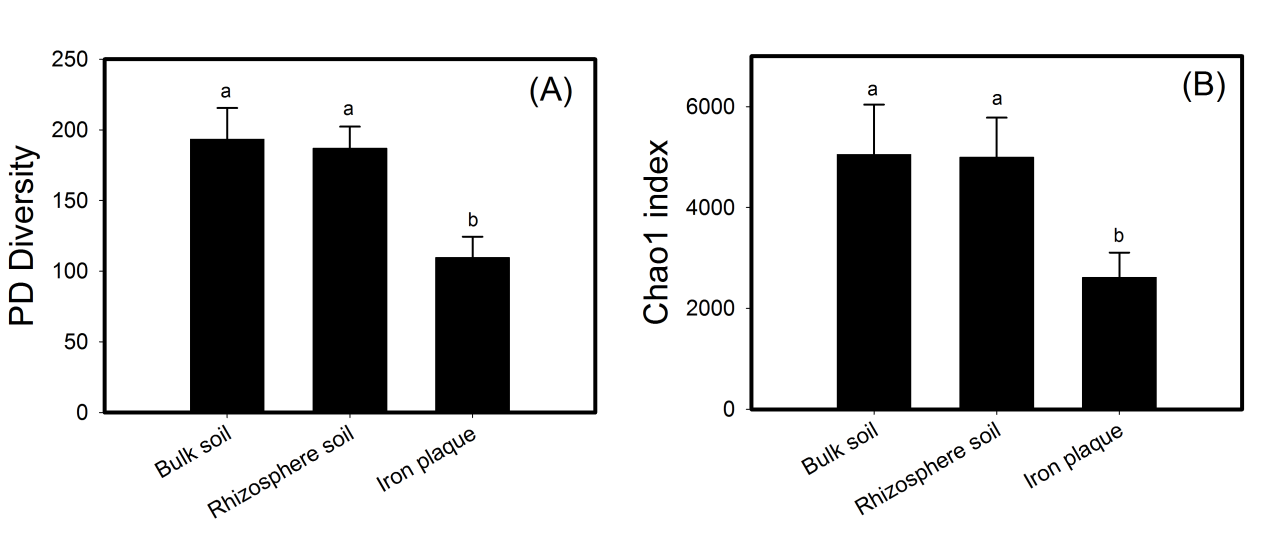


**Figure S1.** The microbial community diversity of bulk soil, rhizosphere soil and iron plaque.


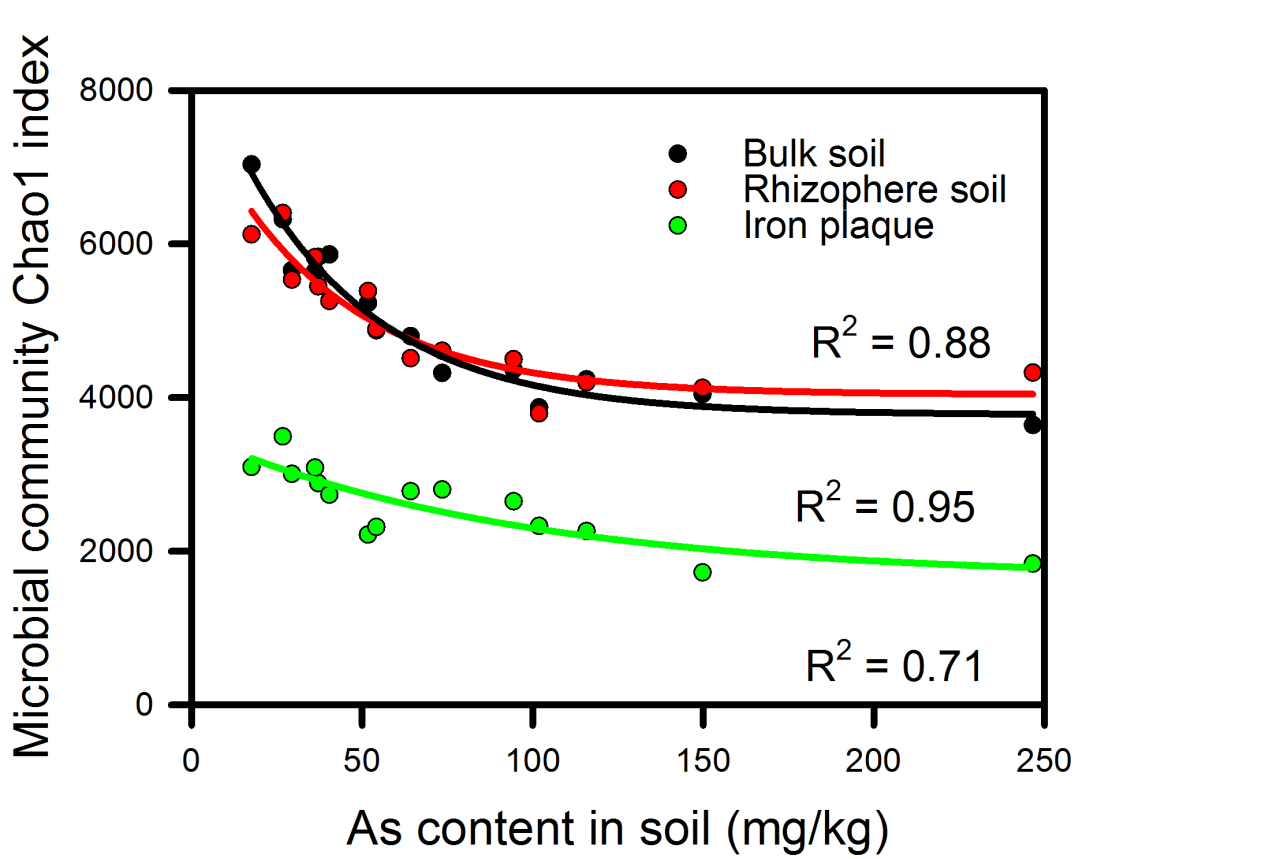


**Figure S2.** The correlation between total As content in bulk and microbial community diversity of bulk soil, rhizosphere soil and iron plaque.


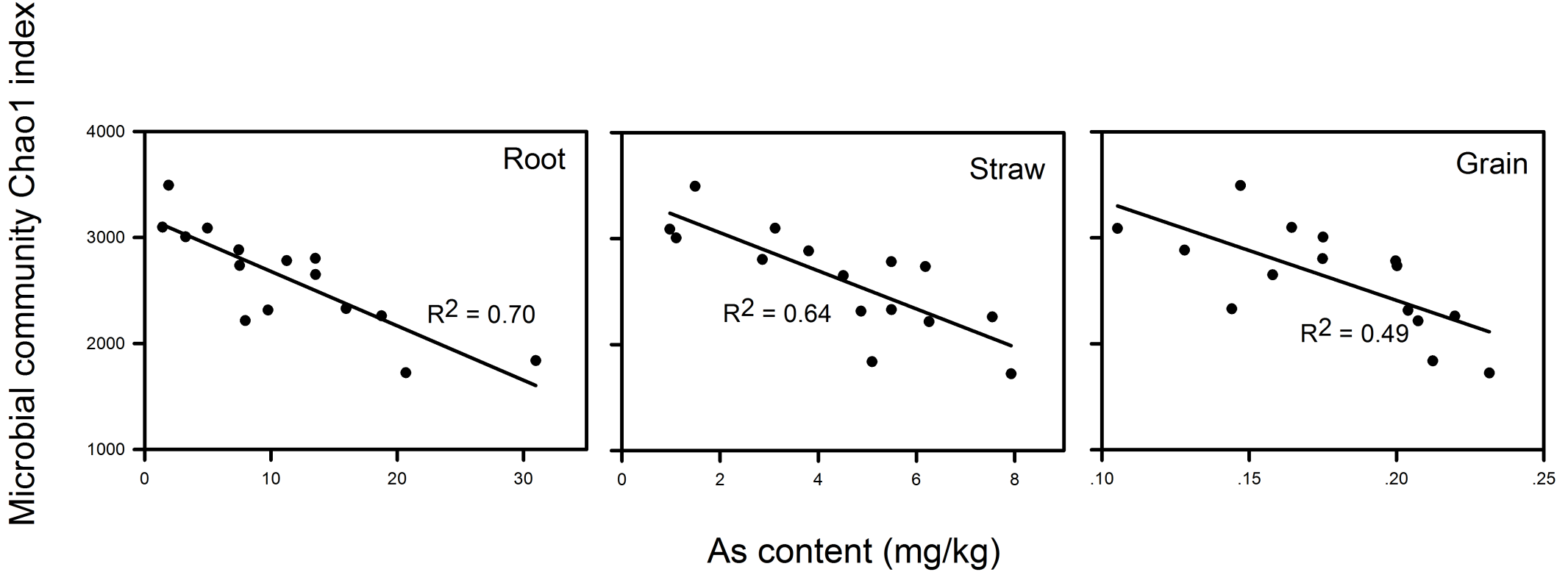


**Figure S3.** The correlation between microbial community diversity of iron plaque and arsenic content in rice root, straw and grain.


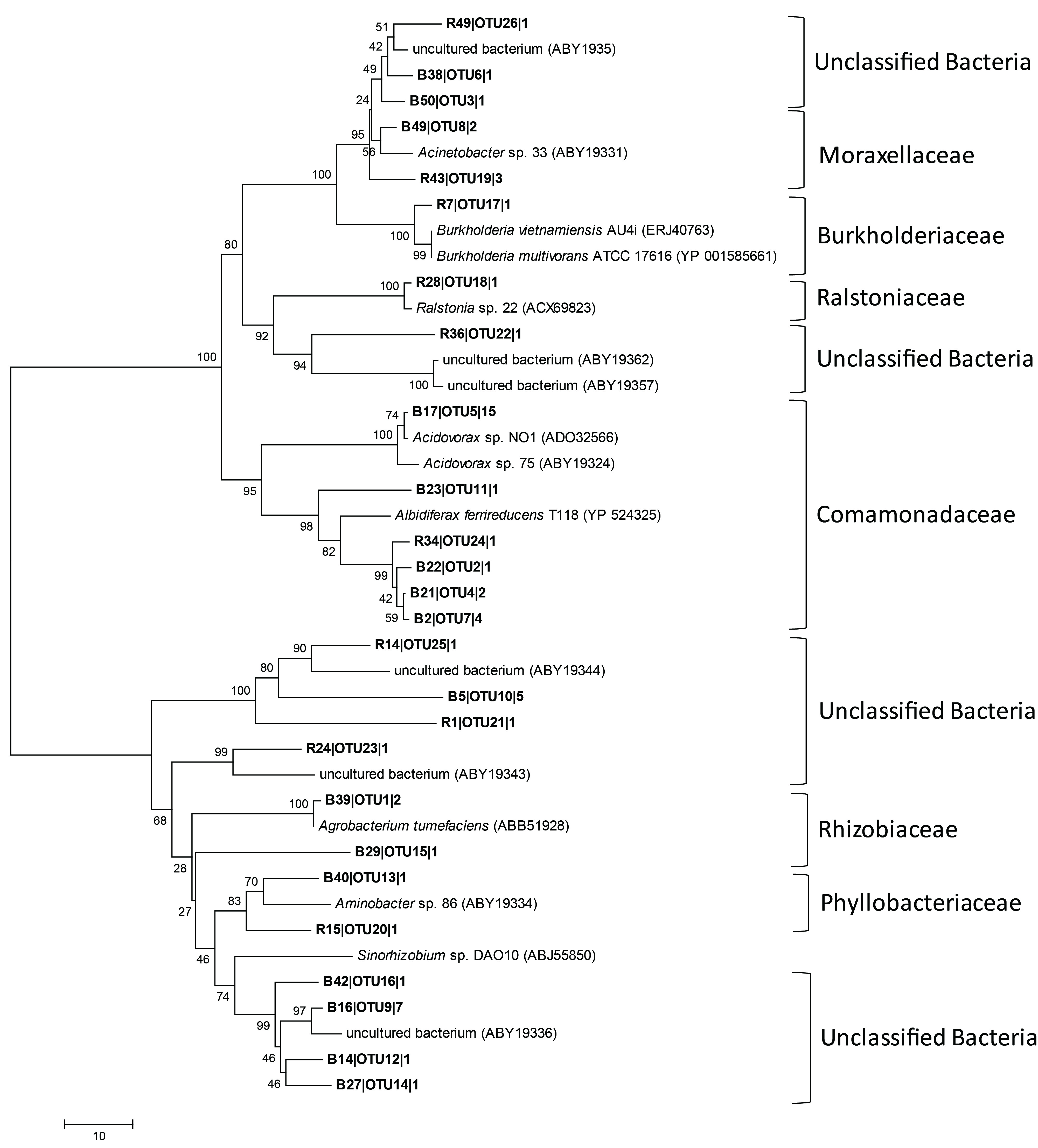
**Figure S4.** Neighbor-joining tree showing the phylogenetic relationship of arsenite-oxidizing bacteria on the root iron plaque.

1. ^*^**Corresponding Author.** Tel.: +86 20 37021396; Fax: +86 20 87024123.

   *E-mail address*: [cefbli@soil.gd.cn](mailto:cefbli@soil.gd.cn) (Dr. F.B. Li) [↑](#footnote-ref-2)
